# Early surgery with antibiotics treatment had better clinical outcomes than antibiotics treatment alone in patients with pyogenic spondylodiscitis: a retrospective cohort study

**DOI:** 10.1186/s12891-017-1533-1

**Published:** 2017-04-27

**Authors:** Tsung-Ting Tsai, Shih-Chieh Yang, Chi-Chien Niu, Po-Liang Lai, Ming-Hsun Lee, Lih-Huei Chen, Wen-Jer Chen

**Affiliations:** 1 Department of Orthopaedic Surgery, Spine Section, Bone and Joint Research Center, Chang Gung Memorial Hospital and Chang Gung University College of Medicine, No. 5, Fusing St., Gueishan, Taoyuan 333 Taiwan; 2 Department of Orthopaedic Surgery, E-Da Hospital, I-Shou University, Kaohsiung, Taiwan; 3 Division of Infectious Diseases, Department of Internal Medicine, Chang Gung Memorial Hospital and Chang Gung University College of Medicine, Taoyuan, Taiwan

**Keywords:** Pyogenic spondylodiscistis, Medical treatment, Antibiotic therapy, Surgical approaches, Kyphosis angle, Oswestry disability index

## Abstract

**Background:**

Pyogenic spondylodiscitis is a form of spinal infection that can result in severe back pain and even death. However, information is lacking on the relative effectiveness of various therapies. A retrospective chart review was conducted to investigate whether early surgical treatment of pyogenic spondylodiscitis coupled with intravenous antibiotics results in better patient prognoses than intravenous antibiotics therapy alone.

**Methods:**

All patients treated for pyogenic spondylodiscitis at a single medical center from July 2006 to July 2011 were retrospectively reviewed. The inclusion criteria consisted of diagnosis of an early stage infection without neurological deficit, and patients without severe sepsis who were suitable candidates for early surgery as determined by a Pittsburgh bacteremia score < 4, and patients with delayed diagnosis and lost to outpatient follow-up were excluded. Clinical outcomes included patient demographic data, kyphosis angle, length of treatment, Oswestry Disability Index and visual analogue pain scale were analyzed.

**Results:**

Of 90 enrolled patients, Group 1 (*n* = 47) received only antibiotic therapy and Group 2 (*n* = 43) received early surgery with post-surgery antibiotics for 2 to 4 weeks. Group 2 exhibited significantly better results than Group 1 for mean antibiotic administration period, mean hospitalization period, kyphotic angle correction. Of 61 patients who participated in telephone follow-up after discharge, Group 2 (*n* = 26) had significant lower mean ODI score, and mean back pain score than Group 1 (*n* = 35).

**Conclusions:**

While infection control was similar for both groups, patients treated with early surgery and antibiotics were hospitalized for fewer days and required less antibiotics than those treated with antibiotics alone, also having better functional outcomes. In short, early surgical treatment of pyogenic spondylodiscitis typically achieves a better prognosis, shorter hospitalization period, and subsequent significant improvement in kyphotic deformity and quality of life.

## Background

The overall incidence rate of spondylodiscitis is increasing as average life-expectancy increases. Moreover, increases in various risk factors (such as intravenous drug use) and medical comorbidities (such as health care-related infections) are contributing to the rising incidence rate for spondylodiscitis. Fortunately, while the incidence rate is gradually rising, advances in microbiological, radiological and surgical techniques have substantially decreased morbidity and mortality rates for the condition [1–3].

Because the symptoms of spondylodiscitis are fairly non-specific, diagnosis can be difficult, and typically does not occur at the earliest stages, when treatment would be most efficacious [4, 5]. When a diagnosis of spondylodiscitis is made, it is usually on the basis of a combination of clinical, laboratory, and radiological indicators, with an initial suspicion typically being based on clinical symptoms including fever, back pain, and spinal deformity, among others. However, one or all of these symptoms may be lacking in some cases of spondylodiscitis, while some, such as back pain, also frequently occur in the general population for completely unrelated reasons. As such, a suspicion of spondylodiscitis based on clinical observations must usually be confirmed by additional testing. A firm laboratory diagnosis is usually made on the basis of elevated levels of C-reactive protein (CRP) and a high erythrocyte sedimentation rate (ESR); a high level of leukocytosis is also diagnostically useful, though to a lesser degree.

Even after the diagnosis of pyogenic spondylodiscitis has been confirmed, the appropriate treatment may not be entirely clear. As indicated above, antibiotic therapy (administered intravenously at first, then orally after the patient’s condition is sufficiently stabilized) is standard treatment for most cases, but it is often unclear whether antibiotic treatment should be combined with surgery or more conservative treatments [6].

A number of factors must be considered when deciding if surgical treatment is warranted, including patient age, overall patient health, and the severity of clinical symptoms such as bone destruction [7]. For example, due to the risks associated with surgery, conservative therapy is often considered the best option for very elderly or weak patients [8]. On the other hand, because long-term immobilization of the spine is an important part of conservative therapy, clinicians must also weigh the dangers of bed rest (e.g., deep vein thrombosis, muscular atrophy, and pneumonia), especially for elderly patients, against the aforementioned risks of surgical treatment.

In short, recommendations relating to the wisdom of treating spondylodiscitis via surgical means (i.e., in addition to antibiotic therapy), remain a source of some controversy [7, 9–11]. In the present study, then, we sought to clarify this controversy by comparing the clinical outcomes of pyogenic spondylodiscitis patients treated with both early surgery and antibiotics who received surgical treatment immediate after confirming the diagnosis of spondylodiscitis at a early stage of infection with no neurological deficit and complications such as sepsis presented with those of patients treated with antibiotics only, and excluded patients treated with delayed surgery who received surgical treatment following the failure of prolonged medical management or delayed diagnosis of pyogenic spondylodiscitis presenting with neurological deficit or spinal instability with intractable back pain.

## Methods

### Patient populations

Ethical approval was obtained from the Institutional Review Board of Chang Gung Medical Foundation (100-4083B). The data for the present study were gathered via retrospective chart review of the records of patients at a single medical center. The time period covered by the chart review ranged from July 2006 to July 2011. The hospital’s records clearly indicated those patients who were diagnosed with pyogenic spondylodiscitis during the period in question. Diagnosis was based on clinical findings, radiological features and laboratory results. For patients who had fever and back pain with clinical suspicion of spinal infection, plain radiographs of the spine were taken initially to screen for any abnormality, and MRI was required to confirm the diagnosis by distinguishing infections from degenerative changes and neoplasms. Furthermore, ESR, CRP and WBC count were evaluated, and blood culture and biopsy (if needed) were performed to identify the bacterial pathogen.

Those patients identified as having spondylodiscitis were then screened for retrospective inclusion of their demographic and clinical data in the current study. The inclusion criteria included the following: patients who were diagnosed with an early stage infection, had no signs of neurological deficit, and had Pittsburgh bacteremia score of no more than 4, which indicating no severe sepsis so patients are suitable candidates for medical treatment and early surgical treatment. The Pittsburgh bacteremia score is a previously validated scoring system based on body temperature, blood pressure, mental status, the need for mechanical ventilation, and the presence or absence of cardiac arrest [12]. Patients were excluded from this study if they lost to outpatient follow-up including clinical and x-ray examinations during 12 months after discharge, received treatment but unfortunately expired during hospital stay, and received medical treatment at first and shifted to surgical treatment due to poor response to antibiotics.

The patients who met the inclusion criteria and were suitable candidates for surgery with similar general health status and medical comorbidities, were then retrospectively divided into two groups according to which type of treatment the patient received depend on his/her willingness to accept risks for surgery: Group 1 consisted of patients who only received antibiotic treatment, and Group 2 consisted of patients who underwent early surgery followed by subsequent treatment with antibiotics. Patients in Group 2 received post-surgery antibiotic therapy for 2 to 4 weeks. In general, empirical antimicrobial therapy was given initially, and then an appropriate antibiotic was administrated based on the results of microbial culture and advice from the infectious disease attending physician. The dosage and treatment duration were based on the weight of the patient, and his/her renal and liver functions. The withdrawal of antibiotic therapy was indicated based on the infection-related laboratory test results, for example, the normal levels of CRP and ESR, and advice from the infectious disease attending physician. The surgical method for each surgery is primarily dependent on surgeons’ preference, by which surgeons chose the approach they are most familiar with the infected site of the spine. All the patients were followed up and evaluated at 12 months post-operatively.

### Outcome measures

The following data of each patient were collected for statistical analysis: gender, age, underlying comorbidity, pre- and post-treatment kyphosis angles (as indicated by X-ray), length of hospital stay, length of antibiotics treatment, tissue culture rate, post-treatment Oswestry Disability Index (ODI) score and post-treatment visual analogue pain scale (VAS) score for back pain [13]. The kyphosis angles were measured by an orthopedic research fellow who was blinded to the grouping method of the patients. Using a picture archiving and communication system (PACS), the angles were measured according to the upper end-plate line of the uppermost and lower end-plate line of the lowermost vertebrae involved. The validated local language version of the Oswestry Disability Index 2.1 was obtained from Dr. Lu [14]. ODI and VAS scores were obtained by post-discharge follow-up via telephone by an orthopedic research fellow who was blinded to the patient grouping method.

### Statistical analysis

Statistical analysis and comparisons were then carried out on the data for both groups of patients using SPSS 21.0 (IBM-SPSS Inc., Chicago, IL, US) in order to determine whether or not there were statistically significant differences between these two groups both before and after treatment. Specifically, chi-square testing was performed to compare the gender, underlying comorbidity, and tissue culture rate distributions between the two groups, while t tests were performed to compare the groups with regard to the following continuous variables: age, mean pre-treatment kyphosis angle, mean kyphosis correction angle, mean length of hospitalization, mean length of antibiotics treatment, mean ODI score after treatment, and mean VAS score after treatment. In addition, an analysis of variance (ANOVA) was carried out to determine whether there were any intragroup differences in the outcomes for the Group 2 patients in terms of the different types of surgeries they received. A p value of < .05 was considered significant for all statistical analyses.

## Results

Data for a total of 90 patients (61 men and 29 women, average age = 60.69 years, SD = 15.14) was deemed suitable for inclusion based on the above criteria. Group 1 (*n* = 47) consisted of 33 men and 14 women with an average age of 62.5 years, and Group 2 (*n* = 43) consisted of 28 men and 15 women with an average age of 58.9 years. Nine patients (7 received medical treatment and 2 received early surgical treatment) who had progressive infection and developed severe sepsis and then subsequently died were excluded from this study; in addition, seven patients who initially met the criteria for Group 1 later required surgery due to poor response to antibiotics were also not included in either group. For the Group 2 patients, transforaminal lumbar or costotransversectomy thoracic approach interbody debridement from the posterior approach and fusion [15, 16] was performed in seven patients, anterior radical debridement and fusion was performed in 14 patients, and an anterior procedure completed with posterior instrumentation and fusion was carried out in 22 patients. Two patients had postoperative deep wound infection from the same bacterial pathogen of discitis and required repeat debridement. The data for the 47 Group 1 patients and the 43 Group 2 patients were analyzed as described above. The patient characteristics are presented in Table 1. As indicated, there were no significant demographic differences between the two groups prior to treatment in terms of age or gender. Likewise, there were no significant differences between the two groups in terms of mean pre-treatment kyphosis angle or comorbidity. To sum up, these two groups were similar across the full range of measured variables prior to treatment.

**Table 1:** Demographic data of patients in the Group 1 and Group 2 treatment groups

	Group 1: Antibiotics only (*n* = 47)	Group 2: Early surgery with antibiotics (*n* = 43)
Gender, *n* (%)		
Male	33 (70.2%)	28 (65.1%)
Female	14 (29.8%)	15 (34.9%)
Age (in years)		
Mean (SD)	62.5 (15.2)	58.9 (15.1)
Range	30–85	16–82
Underlying comorbidity, *n* (%)		
DM	11 (23.4%)	16 (37.2%)
ESRD	1 (2.1%)	4 (9.3%)
Liver cirrhosis	5 (10.6%)	5 (11.6%)
RA	0 (0.0%)	1 (2.3%)
Drug addiction	3 (6.4%)	3 (7.0%)
Pre-treatment kyphosis angle^a^		
Mean (SD)	+11.2 (8.4970)	+10.986 (8.0919)
Range	1.2–35.3	0.6–32

*SD* standard deviation, *DM* diabetes mellitus, *ESRD* end-stage renal disease, *RA* rheumatoid arthritis

^a^Kyphosis angle: unit in degrees; positive value = kyphosis, negative value = lordosis

**Fig. 1:**
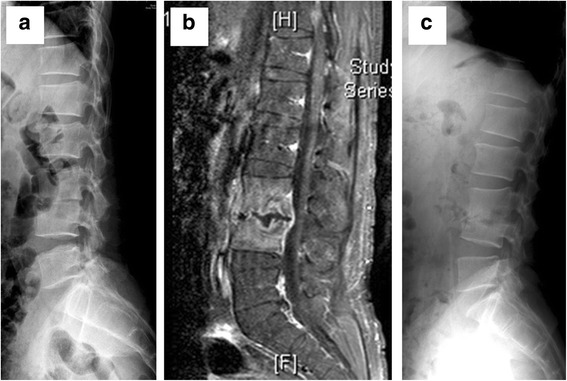
Pre- and post-treatment images of a 38 years old male heroin addict in Group 1. **a** Lateral X-ray view of the spine with pre-treatment kyphosis angle of 8.7°. **b** Sagittal section of contrast MRI shows disc destruction and end-plate erosion, which indicating pyogenic spondylodiscitis at L3/4. **c** Lateral X-ray view after the patient received antibiotics treatment for 42 days with post-treatment kyphosis angle of 13.3°. The patient’s total hospital stay was 47 days, and X-ray revealed progressive kyphosis after the antibiotics treatment

**Fig. 2:**
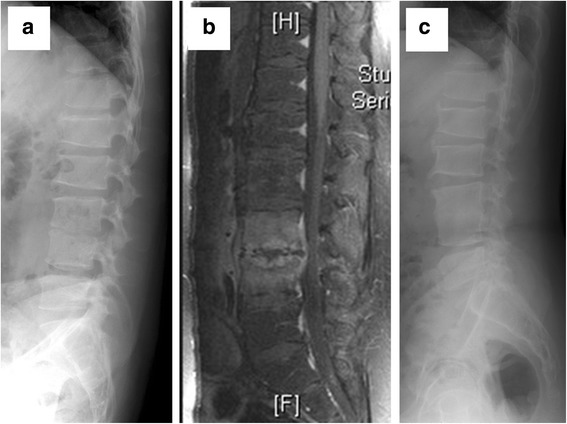
Pre- and post-treatment images of a 33 years old male heroin addict in Group 2. **a** Lateral X-ray view of the spine with pre-treatment kyphosis angle of 3.2°. **b** Sagittal section of contrast MRI shows disc destruction and end-plate erosion, which indicating pyogenic spondylodiscitis at L3/4. **c** Lateral X-ray view with post-treatment kyphosis angle of 1.1°, and it revealed bone union after the patient received early surgery using a retroperitoneal approach and L3/4 interbody debridement and fusion, in addition to antibiotics treatment. The patient’s total hospital stay was 25 days, and kyphosis angle correction (1.1–3.2 = −2.1) revealed an improved kyphosis angle and stable spine after surgical correction

In contrast, there were significant differences between the two treatment groups in terms of a number of outcome measures, as shown in Table 2. The mean antibiotic administration period for the patients who were treated only with antibiotics (Group 1) was 46 days, while the mean antibiotic administration period for the patients who underwent early surgery followed by subsequent treatment with antibiotics (Group 2) was only 31 days (*p* < 0.001). In addition, the mean hospitalization period for Group 1 was 51.2 days, while the mean hospitalization period for Group 2 was 33.4 days (*p* < 0.001). A comparison of the pre- and post-treatment kyphosis angles for the two groups indicated that Group 2 exhibited significantly better improvement in deformity in terms of greater kyphotic angle correction (*p* < 0.05). Examples of this difference in kyphosis angle correction for the two groups are shown via pre- and post-treatment X-rays for two patients, one from each group, Group 1 in Fig. 1 and Group 2 in Fig. 2.

**Table 2:** Outcome measures for patients in the Group 1 and Group 2 treatment groups

	Group 1: Antibiotics only (*n* = 47)	Group 2: Early surgery with antibiotics (*n* = 43)	*P* value
Kyphosis correction angle^a^			
Mean (SD)	+1.2 (5.0)	−2.1 (6.3)	0.024
Length of hospital stay (days)			
Mean (SD)	51.2 (23.2)	33.4 (17.5)	0.0001
Length of antibiotics Tx (days)			
Mean (SD)	46.0 (21.7)	31.0 (16.9)	0.0001
Tissue culture rate, *n* (%)			
Growth	23 (48.9%)	36 (83.7%)	0.001
No growth	24 (51.1%)	7 (16.3%)	

*SD* standard deviation, *Tx* treatment

^a^Kyphosis correction angle = post-treatment kyphosis angle - pre-treatment kyphosis angle

In addition, the two treatment groups also exhibited significant differences in terms of quality of life measures (VAS and ODI) obtained through the telephone follow-up after discharge. However, there were a substantial number of patients in both groups were unwilling or unable to contact by telephone to complete the follow-up questionnaire. As detailed in Table 3, Group 1 (*n* = 26) consisted of 18 men and 8 women with an average age of 62.0 years, and Group 2 (n = 35) consisted of 28 men and 15 women with an average age of 58.9 years. Similarly to Table 1, there were no significant demographic differences between groups in age, gender and pre-treatment kyphosis angle, which suggested that these two groups had similar demographics prior to treatment. There was a significant difference (*p* < 0.05) between the mean ODI scores for the two groups, which were 14.5 and 8.8 for Group 1 and Group 2, respectively. The patients in the two groups also reported significantly different levels of back pain (Group 1 = 1.7, Group 2 = 0.8; *p* < 0.05) as measured by VAS. Taken together, these results suggest that the patients who received early surgery in addition to antibiotics treatment achieved significantly better quality of life than those who were only treated with antibiotics.

**Table 3:** Quality of life outcome measures in the Group 1 and Group 2 treatment groups at 12-month follow-up

	Group 1: Antibiotics only (*n* = 26)^b^	Group 2: Early surgery with antibiotics (*n* = 35)^b^	*P* value
Gender, *n* (%)			
Male	18 (69.2%)	26 (74.3%)	0.663
Female	8 (30.8%)	9 (25.7%)	
Age (in years)			
Mean (SD)	62.0 (13.8)	58.4 (14.8)	0.348
Range	30–85	16–82	
Pre-treatment kyphosis angle^a^			
Mean (SD)	+11.765 (8.4780)	+11.071 (7.2817)	0.741
Range	1.2–35.0	1.7–29	
VAS score for back pain			
Mean (SD)	1.7 (1.7)	0.8 (1.4)	0.034
ODI score			
Mean (SD)	14.5 (12.9)	8.8 (8.4)	0.048

*VAS* visual analogue pain scale, *ODI* Oswestry Disability Index

^a^Kyphosis angle: unit in degrees; positive value = kyphosis, negative value = lordosis

^b^A substantial number of patients in both groups were unwilling or unable to contact by telephone to complete the follow-up questionnaire used to assess VAS and ODI after discharge

In summary, the patients who underwent early surgery followed by treatment with antibiotics had significantly better outcomes across a number of measures than those patients who only received treatment with antibiotics. Meanwhile, it should also be noted that according to a within-group ANOVA, there were no significant differences in the outcomes for patients within Group 2 in terms of which of the three types of surgeries they received, namely, debridement and fusion from the posterior approach, anterior radical debridement and fusion, and anterior procedure completed with posterior instrumentation, which meaning that the outcomes for the different surgery types can be viewed collectively for comparison with the outcomes for Group 1, the non-surgery treatment group.

## Discussion

Pyogenic spondylodiscitis has generally been regarded as medical disease requiring antibiotics treatment as a first resort [3]. Surgery, in contrast, has historically been recommended only under one or more of the following conditions: the compression of neural elements, spinal instability due to extensive bony destruction, severe kyphosis, or the failure of non-surgical management [17–19]. In neurologically intact patients, the more conservative, non-surgical approach has increasingly been used with success and has been advocated provided a microbiological diagnosis is available. Patients treated with antibiotics have no risk of surgery-related complications; however, prolonged antibiotic treatment may not be effective, and also some degree of vertebral body destruction, nerve roots impingement, progressive kyphosis and prolonged back pain may still occur after successful treatment. Early surgery is able to achieve infection control and an immediate pain relief in advance of extensive vertebral destruction leading to spinal instability and kyphotic deformity, but patients may suffer from procedure-related complications. In previous studies, most of the surgical treatments were performed in cases of patients with complications and poor response to a conservative treatment, which make it difficult to compare.

The present study is the first to provide direct comparisons of patient outcomes for an early stage of pyogenic spondylodiscitis patients receiving treatment with antibiotics only versus those receiving antibiotics treatment coupled with early surgical intervention, which early pyogenic spondylodiscitis was defined as neither neurological deficit nor complications such as sepsis was present. Notably, the outcomes for patients who received the surgical treatment included improved functional results, better kyphosis correction, and shorter hospital stays, findings that call into question the validity of the non-surgical approach to treatment that has heretofore been generally accepted. Based on our results, we suggested all patients who are at an early stage of pyogenic spondylodiscitis and are suitable for surgery should be treated surgically immediately after the confirmation of diagnosis to achieve better outcomes; however, conservative treatment is indicated if the patient is not suitable for surgery.

The reasons for the improved outcomes in the surgery-with-antibiotics group most likely reflect the nature of the disease itself. At base, the most important issues in the treatment of pyogenic spondylodiscitis are the infection itself and the spinal instability resulting from it. Undoubtedly, antibiotics are effective for treating the infection; however, they may be relatively ineffective for the purposes of correcting spinal instability. Surgery, in contrast, can be used to remove debris tissues and provide enhanced spinal stability, as well as for obtaining an accurate bacterial culture pathogen. Thus it is that the combination of surgery and antibiotics may often result in better outcomes than treatment with antibiotics alone, insofar as the combination provides treatment of both the infection and the spinal instability at the same time.

Having said that, the current study does have some limitations which call for caution in the interpretation of its results. First, this was a retrospective study. As such, the decisions regarding which treatment to pursue were not random. Moreover, a substantial number of patients in both groups were unavailable for assessing the quality of life outcome, which resulted in performing analyses with two different study populations. This might introduce a selection bias, whereas there was a possibility that patients with low pain and disability scores might have chosen to not participate in the telephone follow-up since they were not suffering from any symptoms, especially in the medical treatment group. A second potential problem relates to the stage of the spinal infection. Simply put, it is difficult to define an “early stage” infection when it comes to pyogenic spondylodiscitis, as there is as yet no generally accepted staging system for this type of infection. Also, there are many confounding factors such as infection degree, treatment duration and surgical approach may affect the results. However, it is very difficult to have a sufficient number of patients to analyze these factor, since the incidence of pyogenic spondylodiscits is rare and each surgeon has his own preferred approach.

## Conclusions

Even with the aforementioned caveats in mind, however, the results of the present study are compelling enough to suggest that the option of early surgical intervention should be utilized more often. In short, while the end results were similar for both treatment groups in terms of infection control, early surgical intervention can be recommended on the basis of shortened hospital stays and improved quality of life, as determined by both the pain and disability levels reported at the 12-month follow-up.

At the same time, future research should be conducted to ameliorate some of the present study’s limitations and provide clarity regarding its findings. First, a prospective random control study should be conducted to eliminate the potential selection biases noted above. Second, a staging system for pyogenic spondylodiscitis should be developed for the purposes of improving the comparison of the different treatment methods. Finally, a protocol should be developed, based on the results of the present and subsequent studies, to aid physicians in deciding when to use surgical intervention, as well as to determine which specific surgical intervention is most suitable for a given kind of infection.

## Abbreviations

CRP: C-reactive protein
DM: Diabetes mellitus
ESR: Erythrocyte sedimentation rate
ESRD: End-stage renal disease
ODI: Oswestry disability index
PACS: Picture archiving and communication system
RA: Rheumatoid arthritis
SD: Standard deviation
VAS: Visual analogue pain scale
